# The invisible magnitude of the rape of girls in Brazil

**DOI:** 10.11606/s1518-8787.2021055003439

**Published:** 2021-12-01

**Authors:** Stella Regina Taquette, Denise Leite Maia Monteiro, Nádia Cristina Pinheiro Rodrigues, José Augusto Sapienza Ramos

**Affiliations:** I Universidade do Estado do Rio de Janeiro Faculdade de Ciências Médicas Departamento de Pediatria Rio de Janeiro RJ Brasil Universidade do Estado do Rio de Janeiro. Faculdade de Ciências Médicas. Departamento de Pediatria. Rio de Janeiro, RJ, Brasil; II Universidade do Estado do Rio de Janeiro Faculdade de Ciências Médicas Departamento de Ginecologia e Obstetrícia Rio de Janeiro RJ Brasil Universidade do Estado do Rio de Janeiro. Faculdade de Ciências Médicas. Departamento de Ginecologia e Obstetrícia. Rio de Janeiro, RJ, Brasil; III Fundação Oswaldo Cruz Escola Nacional de Saúde Pública Rio de Janeiro RJ Brasil Fundação Oswaldo Cruz. Escola Nacional de Saúde Pública. Rio de Janeiro, RJ, Brasil; IV Universidade do Estado do Rio de Janeiro Instituto de Medicina Social Rio de Janeiro RJ Brasil Universidade do Estado do Rio de Janeiro. Instituto de Medicina Social. Rio de Janeiro, RJ, Brasil; V Universidade do Estado do Rio de Janeiro Núcleo de Geotecnologias Rio de Janeiro RJ Brasil Universidade do Estado do Rio de Janeiro. Núcleo de Geotecnologias. Rio de Janeiro, RJ, Brasil; VI Centro Universitário Serra dos Órgãos UNIFESO Departamento de Ginecologia e Obstetrícia Teresópolis RJ Brasil Centro Universitário Serra dos Órgãos (UNIFESO). Departamento de Ginecologia e Obstetrícia. Teresópolis, RJ, Brasil

**Keywords:** Rape, Mandatory Reporting, Underregistration, Child Abuse, Sexual, Pregnancy in Adolescence

## Abstract

**OBJECTIVE:**

Compare official data on notifications of sexual violence against girls aged 10 to 13 years with data on pregnancy for the same age group between 2012 and 2018.

**METHODS:**

This is an epidemiological, descriptive, cross-sectional study with data from the Department of Informatics of the Unified Health System (DATASUS) on violence against and pregnancy of girls aged 10 to 13 years from 2012 to 2018. Data on sexual violence were accessed in the Health Information System (SINAN); on pregnancy, in the Live Births Information System (SINASC), on fetal deaths, in the Mortality Information System (SIM), and on abortions, in the Hospital Admission System (SIH).

**RESULTS:**

Between 2012 and 2018, out of 136,387 pregnancies, there were 120,185 live births and 15,402 interrupted pregnancies by abortions or fetal deaths of mothers who became pregnant aged 13 years or younger. In the same period, SINAN received 46,548 notifications of sexual abuse against girls aged 10 to 13 years. The number of girls who became pregnant before the age of 14, victims of statutory rape, was 2.9 times higher than the number of cases notified to SINAN.

**CONCLUSION:**

The lack of adequate notification of statutory rapes in Brazilian official statistics leads to the underestimation of its magnitude.

## INTRODUCTION

Sexual violence is a prominent crime in Brazil, but there is little evidence of it in official statistics. According to the latest national victimization survey, only 10% of victims report the assault to police authorities^1^, and estimates set notifications to SINAN three times as low^2^. Girls under 14 years of age are the most frequent victims of sexual offenses. Of all cases notified in 2017 and 2018, more than half (53.6%) of the victims were under 14 years of age; of these, most were females aged 10 to 13 years (81.8%). The situation worsened in 2019, with 66.348 reported cases in which 57.9% of the victims were up to 13 years old. Among these, 85.7% were female^3^, showing an 8% increase in relation to previous data; an incidence rate of four girls (up to 13 years old) per hour in Brazil^2^.

Several studies corroborate the official statistics, showing that black girls in this age group comprise most victims^4,5^. Some factors are significant for this statistic, such as gender inequality produced by a patriarchal culture, and the vulnerabilities inherent to this age group, such as economic dependence, reduced autonomy, low schooling, and ongoing growth and development^6^. Where social inequality and poverty are greater, the sexual exploitation of minors is an important factor affecting sexual violence rates^9,10^.

The Brazilian penal legislation since 2009 defines statutory rape as intercourse or other libidinous acts committed against minors under 14 of age^11^, and though its report is compulsory, we must emphasize that notifications are low^1^. Sexual violence is often not perceived - let alone informed - because it occurs more often in victims’ homes via seduction, coercion, and/or threats committed by a family member or acquaintance^2,12^, evidencing its under-notification and the failure of official indices in portraying the reality of sexual violence against adolescents in this age group. Other forms of violence may also be less visible due to the absence of notifications, such as marital sexual violence^15^.

Reasons for the non-notification of violent crimes are fear of the aggressor’s retaliation, the trial after the accusation, institutional discredit, etc.^2^. Another hypothesis claims the under-notification of statutory rapes is due to victims and their families’ dismissing it as violence^16^, normalizing it if related to the trend for early sexual initiation in Brazilian adolescents, often before the age of 14^17,18^, as the high pregnancy rates in this group show. These rates are decreasing in recent years, but remain high compared to developed countries^19^ and, in the age group between 10 to 13 years, continue to rise in the North and Northeast of Brazil^20^.

In view of the data, we emphasize that pregnant girls between 10 and 13 years of age are, according to the law, victims of statutory rape, and official statistics might ignore this. Since it occurs mainly in a private environment, some factors contribute to non-notification, such as families concealing the crime, victims’ vulnerable situation, or even the difficult access to a legal public abortion service^21^, meaning these deliveries will elude official statistics on sexual violence.

This study aims to compare official data on the notification of sexual violence against girls aged 10 to 13 years with data on pregnancy for the same age group, corresponding to statutory rape, between 2012 and 2018. We aim to contribute to the reduction of the information gap on statutory rape in Brazil and, consequently, with the implementation of measures to protect potential victims.

## METHODS

This is an epidemiological, descriptive, cross-sectional study, developed with DATASUS data on sexual violence against and pregnancy of girls aged 10 to 13 years between 2012 to 2018. Data on sexual violence were accessed in the SINAN databases on violence^a^; on adolescent pregnancy, in the Live Birth Information System (SINASC)^b^; on fetal deaths, in the Mortality Information System (SIM)^c^; and on abortion, in the *Sistema de Informações Hospitalares do SUS* (SUS Admission Information System - SIH), Chapter XV of the ICD-10 (pregnancy, childbirth and puerperium) and the Morbidity list – miscarriage, abortion for medical reasons, and other pregnancies ending in abortion^d^.

DATASUS is managed by the Secretariat of Health Surveillance along state and municipal health departments, and data are grouped by age from 10 to 14 years. These institutions collect live birth declarations from health services and registries (for home births) and insert the data into the SINASC. To estimate the data for mothers aged 10 to 13 years, the following searches and estimations were performed. For each year and federative unit, a dBase File Compacted (*.dbc) file is provided; converted to a dBase File (*.dbf) via batch script (.bat) in the TabWin application, developed by DATASUS^e^. Since the assembled database had 68.8 million records between 1994 and 2018, a database management system (DBMS) was necessary to analyze and manipulate the large volume of data. PostgreSQL, version 11.8 was chosen for that. Data were imported into PostgreSQL from .dbf files via scripts developed in Python version 3.8. As SINASC records a priori all live births (LB) in Brazil, two groups from the database were selected for this study:

Group 1: pregnant women aged 10 to 13 years who had LBs between 2012 to 2018. Mothers were selected by their age at childbirth.

Group 2: adolescents whose pregnancies began at the age of 13, ending in a live birth at 14 between 2012 and 2018. This selection estimated the difference between mothers’ and gestational ages (in weeks) at birth.

Although the SINASC database ranged from 1994 to 2018, Group 1 was comprised only of records from 2012 onwards, since the system was being tested and implemented in Brazil between 1994 and 1995^22,23^. Moreover, SINASC register fields changed over time, most substantially probably between 2010 and 2012^f^. From 2012 onwards, more than 90% of LB records had the *semagestac* attribute to estimate the mother’s age at the beginning of pregnancy for Group 2. Thus, the period from 2012 to 2018 was selected.

The number of SINASC records characterizing the statutory rape of a minor under 14 years of age, according to Art. 217-A of Federal Law N^o^ 12.015 of August 7, 2009^11^is obtained by adding the incidents for Group 1 with Group 2. The fertility rate by specific age (FRSA‰) was estimated via the number of LBs whose mothers became pregnant aged 10 to 13 years per 1,000 female adolescents in the same age group, distributed by federative unit.

Data provided by SINAN, SIM and SIH also refer to the same age group. To obtain an estimate of the number of cases of violence, fetal deaths, and abortions in this age group, the projection from the Population Census of the Brazilian Institute of Geography (IBGE) was used^24^. The proportion of the female population aged 10 to 13 years in the total population of the same age and gender was estimated, and a value of 0.79 was found for every year between 2012 and 2018. That is, 79% of the total group aged 10 to 14 years is comprised of females aged 10 to 13 years. Thus, the number of fetal deaths, abortions and notifications of sexual violence against females aged 10 to 13 years was estimated by multiplying the total female population aged 10 to 14 years by 0.79.

## RESULTS

Between 2012 and 2018, there were 58,922 notifications of sexual violence against female victims aged 10 to 14 years, of which an estimated 46,548 cases were against girls aged 10 to 13 years. We observed a gradual increase in case notifications in all Brazilian federative units in this period, despite the small reduction in some of them in 2015. The Southeast is the federative unit with most notifications. However, when we estimated the rates of sexual violence by the population of each federative unit, we observed that for all years, the North had the highest notification rate per 100,000 female inhabitants aged 10 to 13 years, followed by the South. (Table 1).

**Table 1:** Distribution of the number of cases of sexual violencea notified by SINAN and the rate per 100.000 inhabitants in females aged 10 to 13 years, by federative unit between 2012 to 2018.

Year	North n (Tx/100,000)	Northeast n (Tx/100,000)	Midwest n (Tx/100,000)	Southeast n (Tx/100,000)	South n (Tx/100,000)
2012	1,164 (169.48)	713 (35.66)	421 (87.42)	1,457 (57.87)	875 (101.63)
2013	1,508 (221.83)	1,084 (55.48)	519 (109.09)	1,555 (63.49)	1,070 (128.52)
2014	1,695 (252.17)	1285 (67.31)	507 (107.73)	1,615 (67.67)	1,109 (137.33)
2015	1,560 (234.28)	1,127 (60.19)	615 (131.41)	1,683 (72.05)	1,139 (144.41)
2016	1,671 (252.65)	1,208 (65.54)	684 (146.57)	2,004 (87.68)	1,243 (161.54)
2017	1,778 (270.01)	1,427 (78.56)	811 (174.76)	2,538 (112.83)	1,458 (193.33)
2018	1,922 (292.67)	1,699 (95.03)	880 (191.18)	2,782 (125.23)	1,741 (234.43)
Total	11,298 (241.35)	8,543 (64.81)	4,437 (134.98)	13,634 (82.91)	8,635 (155.42)

^a^ The compulsory form to report cases of violence to SINAN identify the following as sexual violence: sexual harassment, rape, indecent assault, child pornography, sexual exploitation, and others.

According to DATASUS, mothers aged 10 to 14 years delivered 178,622 live births in total within the period, in gradually decreasing annual rates. Table 2 shows the live births for Group 1 — comprised of mothers who delivered under 14 years of age, Group 2 — mothers who became pregnant at 13 years old, but delivered at 14 years old, and the sum of both groups — corresponding to all mothers who conceived aged 13 years and younger. Note the reduction in the estimated annual number of pregnancies for this age group, from 18,348 in 2012 to 14,396 in 2018.

**Table 2:** Annual distribution of deliveries by girls who conceived between 10 and 13 years of 2012-2018.

Year	Mother < 14 years at conception and birth (Group 1)	Mother < 14 years only at conception (Group 2)	Mother who became pregnant aged 10 to 13 years (Group 1 + 2)
2012	6,671	11,677	18,348
2013	6,491	12,417	18,908
2014	5,832	13,008	18,840
2015	5,828	12,180	18,008
2016	5,565	10,790	16,355
2017	4,986	10,244	15,230
2018	4,882	9,614	14,496
TOTAL	40,255	79,930	120,185

Table 3 shows the total number of live births/FRSA‰ for all girls who conceived between 10 and 13 years old (Group 1 and Group 2), distributed across Brazilian federative units. The North and Northeast had the highest rates. Comparing the years in the series show an annual reduction of live births in all federative units, except for a slight increase in the North in 2018.

**Table 3:** Distribution of the number of LB of girls who became pregnant between 10 and 13 years (Group 1 + Group 2) and FRSA‰a by region between 2012 and 2018.

Year	North n (FRSA‰)	Northeast n (FRSA‰)	Midwest n (FRSA‰)	Southeast n (FRSA‰)	South n (FRSA‰)
2012	3,280 (4.88)	6,962 (4.12)	1,613 (3.2)	4,695 (1.87)	1,798 (2.09)
2013	3,446 (5.18)	7,032 (4.25)	1,660 (3.34)	4,947 (2.03)	1,823 (2.2)
2014	3,321 (5.05)	6,882 (4.24)	1,693 (3.44)	5,054 (2.12)	1,890 (2.35)
2015	3,248 (5.0)	6,864 (4.3)	1,576 (3.2)	4,651 (2.0)	1,669 (2.13)
2016	3,132 (4.83)	6,223 (3.94)	1,488 (3.02)	4,077 (1.77)	1,435 (1.86)
2017	2,874 (4.45)	5,942 (3.82)	1,362 (2.79)	3,802 (1.68)	1,250 (1.65)
2018	2,914 (4.52)	5,714 (3.74)	1,272 (2.63)	3,506 (1.57)	1,090 (1.46)
Total	22,215 (4.84)	45,619 (4.05)	10,664 (3.08)	30,732 (1.86)	10,955 (1.96)

^a^ Live births (LB) of mothers who became pregnant between 10 and 13 years by 1000 female adolescents in the same age group.

According to SIM and SIH, from 2012 to 2018, interrupted pregnancies, fetal deaths, and hospital abortions (miscarriages, for medical reasons, and other causes) in our sample totaled 19,497 (3,290, 3,167, 3,282, 2,824, 2,545, 2,364, 2,025, respectively). The estimated total number of interrupted pregnancies in girls aged 10 to 13 years is 15,402 for the same period. Table 4 shows its annual distribution.

**Table 4:** Notification reason, annual distribution of cases of statutory rape, and notifications reported to SINAN in Brazil from 2012 to 2018.

Year	Live Births	Abortions and fetal deaths	Total statutory rapes	Notifications of sexual violence	Reason^a^
2012	18,348	2,599	20,947	4,631	4.5
2013	18,908	2,502	21,410	5,736	3.7
2014	18,840	2,593	21,433	6,211	3.5
2015	18,008	2,231	21,039	6,124	3.4
2016	16,355	2,010	18,365	6,810	2.7
2017	15,230	1,867	17,097	8,012	2.1
2018	14,496	1,600	16,096	9,024	1.8
**TOTAL**	**120,185**	**15,402**	**135,587**	**46,548**	**2.9**

^a^ Ratio between the number of pregnant girls aged 10 to 13 years (statutory rapes) and the number of cases of sexual violence notified in the same age group in females.

Table 4 shows the estimate of statutory rapes and their relationship with cases notified to SINAN. It shows the data on LBs whose mothers became pregnant before the age of 14, and interrupted pregnancies; the sum of which corresponds to the total number of girls who became pregnant before the age of 14 despite the outcome of the gestation, i.e., the total number of victims of statutory rape, according to the Brazilian penal code, and the number of notifications of sexual violence (SV) against females adjusted for the age group. The last column of the table shows the reason for the non-notification. Note that this ratio must be extremely underreported, since many cases remain unnotified in the absence of pregnancies, or if unrecognized as such by victims and their guardians.

## DISCUSSION

This study shows that official statistics fail to reflect the extent of statutory rape in Brazil, since pregnancies of girls aged 10 to 13 years was almost three times as high than the notified cases of sexual violence against girls in the same age group between 2012 and 2018. Our results, however, are less alarming than the ones from a comparative study among pregnant adolescents under 14 years of age. The authors analyzed notified and non-notified violence to the competent agencies between 2011 to 2015, and found that SINAN notifications comprised only 4.3% of 31,611 live births of mothers up to 13 years of age^12^.

SINAN records show notifications increased in all federative units over the years studied, which may evidence a rise in this serious public health problem, and/or an improved notification system. The North had the highest rate of notifications of sexual violence against girls aged 10 to 13 years within the period, and the South, the second highest. However, note that (under)notification of cases of sexual violence must vary by federative unit, since the Northeast had the second highest FRSA average for girls aged 10 to 13 years from 2012 to 2018 — only lower than the North — and the lowest rate of notifications of sexual violence.

A study conducted by Gaspar et al (2018)^25^ on the evolution of notifications of sexual violence in Brazil between 2009 and 2013 showed progress mainly in cases of rape, domestic rape, and repeated sexual violence. The authors claim the number of cases increased, as did notifications due to greater awareness. Victims sought care, health care providers aided them, and notification systems improved. Note also the 2009 change in the penal code which defined statutory rape. From then on, a larger set of actions mobilized both the government and society to face this problem, which may have positively influenced the notification system^26^. On the other hand, another study conducted via records of live births of mothers aged 10 to 13 years in northeastern Brazil evidenced underreporting. The authors found that notifications of sexual abuse comprised only 1.3% of births^27^.

Pregnant minors under 14 years of age, which the law considers cases of statutory rape, and their underreporting to SINAN shows the hidden face of the statistics on sexual violence in Brazil, not to mention on its other forms against this age group which, due to the absence of pregnancies, remain unnotified. When we reflect on the results and the reasons for underreporting, one hypothesis suggests that these pregnancies may be the result of sexual relations which adolescents and their families consider consensual. We know that a considerable percentage of adolescents under 14 years of age are sexually active, often with the knowledge and approval of their families, which sometimes results in marriage. A study conducted with high school students on their perception of sexual initiation and violence showed a disconnection between what adolescents think and what the law provides. We need to broaden the debate on the subject, letting the target audience participate, and intensifying sexual education programs for young people^16^. Note also that aggressors are often families’ relatives or acquaintances, thereby making it difficult for families and healthcare providers to report these cases^2,14^.

We highlight the importance of notifying cases of statutory rape to protect victims and judicially punish aggressors. Healthcare providers are legally obliged to report such cases, but occasionally feel that they should refrain from doing so due to the consensual nature of the sexual relationship, or the barriers they would face in the absence of a well-structured support network. Another datum contributing to non-notification is healthcare providers’ fear of retaliation, due to the idea that the record is an accusation against the aggressor, rather than an initiative to protect the victim^16,27^. Therefore, we need to train these professionals to deal with the subject, including managing family relationships and notifying cases to child protective services.

Sexual violence is a matter of public health and a violation of human rights. The consequences of rape for the life of a teenager are serious and multiple. They need immediate prophylaxis of sexually transmitted infections, HIV, and pregnancy prevention. Problems related to mental health, self-esteem, sociability, growth, and development are prominent in the medium and long terms^28^. The turn into aggressors, increased risk of alcohol and drug use, unprotected sexual activity, and commercial sexual exploitation are also common^29^.

Finally, we emphasize that since pregnancies taking place before the age of 14 are crimes, they entitle the victim to legally terminate the pregnancy. However, studies and the recently televised event of a 10-year-old girl who became pregnant after her uncle had raped her show how difficult it is to guarantee this right for these girls, for them to be recognized as victims of sexual violence^30,31^. This case is emblematic because it highlights an additional barrier to protecting victims of sexual violence in Brazil.

This study is limited because it uses only secondary data, restricted to girls who became pregnant before the age of 14; but it contributes to their visibility. We can infer that the number of girls under 14 years of age suffering sexual violence is much higher than what the notifications suggest, due to their absence even in cases treated in hospitals. Moreover, the *Atlas da Violência* (Atlas of Violence) confirms that less than half of the victims who report to the police seek care in health units^2^. Other sectors responsible for protecting children and adolescents, such as education, child protective services, the Public Council’s Office, and the Public Prosecutor’s Office should participate more effectively in the structure of assistance to adolescents victims of violence, and in the reduction of underreporting and violence itself.

We conclude that the non-notification of cases of statutory rape is one face of this serious and chronic public health problem. Inadequate reporting of statutory rapes in Brazilian official statistics leads to the underestimation of its magnitude. Improving the notification system of sexual violence and guaranteeing the right to protect its victims require public policies aimed at them, especially those in greater individual and social vulnerability with lower access to health services, such as girls under 14 years of age.

## Footnotes

a Ministério da Saúde (BR), DATASUS –Notificação de violência sexual sexo feminino de 10 a 14 anos. Brasília, DF [cited 2020 Sep 13]. Available from: http://tabnet.datasus.gov.br/cgi/tabcgi.exe?sinannet/violencia/bases/violebrnet.def

b Ministério da Saúde (BR), DATASUS. Transferência de Arquivos do SINASC. Brasília, DF [cited 2020 Jul 13]. Available from: http://www2.datasus.gov.br/DATASUS/index.php?area=0901&item=1&acao=28&pad=31655

c Ministério da Saúde (BR), DATASUS. Informações de Saúde (TABNET). Estatísticas vitais: óbitos fetais. Brasília, DF [cited 2020 Oct 31]. Available from:- http://tabnet.datasus.gov.br/cgi/deftohtm.exe?sim/cnv/fet10uf.def

d Ministério da Saúde (BR), DATASUS. Morbidade Hospitalar do SUS – por local de residência – Internações por Ano de atendimento segundo Região. Lista Morb CID-10: Outras gravidezes que terminam em aborto. Faixa etária 1: 10 a 14 anos. Período: 2012. [cited 2020 Oct 31]. Available from: http://tabnet.datasus.gov.br/cgi/tabcgi.exe?sih/cnv/nruf.def

e Ministério da Saúde (BR), DATASUS. Transferência de Arquivos do SINASC. Brasília, DF [cited 2020 Jul 13]. Available from: http://www2.datasus.gov.br/DATASUS/index.php?area=060805&item=3

f Ministério da Saúde (BR), Secretaria de Vigilância em Saúde, Coordenação Geral de Informações e Análises Epidemiológicas. Consolidação do Sistema de Informações Sobre Nascidos Vivos – 2011. Brasília, DF; 2013 [cited 2020 Jul 13]. Available from: http://tabnet.datasus.gov.br/cgi/sinasc/Consolida_Sinasc_2011.pdf
