# Supplementary material for: The invisible magnitude of the rape of girls in Brazil
Source: Rev Saude Publica. 2021 Dec 1;55:103. doi: 10.11606/s1518-8787.2021055003439 (PMC8664051; doi:10.11606/s1518-8787.2021055003439)
Supplement: Translation

# Translation: A invisibilidade da magnitude do estupro de meninas no Brasil

**Authors:** Stella Regina Taquette, Denise Leite Maia Monteiro, Nádia Cristina Pinheiro Rodrigues, José Augusto Sapienza Ramos

**Affiliations:** I Brasil Universidade do Estado do Rio de Janeiro. Faculdade de Ciências Médicas. Departamento de Pediatria. Rio de Janeiro, RJ, Brasil; II Brasil Universidade do Estado do Rio de Janeiro. Faculdade de Ciências Médicas. Departamento de Ginecologia e Obstetrícia. Rio de Janeiro, RJ, Brasil; III Brasil Fundação Oswaldo Cruz. Escola Nacional de Saúde Pública. Rio de Janeiro, RJ, Brasil; IV Brasil Universidade do Estado do Rio de Janeiro. Instituto de Medicina Social. Rio de Janeiro, RJ, Brasil; V Brasil Universidade do Estado do Rio de Janeiro. Núcleo de Geotecnologias. Rio de Janeiro, RJ, Brasil; VI Brasil Centro Universitário Serra dos Órgãos (UNIFESO). Departamento de Ginecologia e Obstetrícia. Teresópolis, RJ, Brasil

**Keywords:** Estupro, Notificação de Abuso, Sub-Registro, Abuso Sexual na Infância, Gravidez na Adolescência

## Abstract

**OBJETIVO:**

Comparar dados oficiais notificados de violência sexual contra meninas de 10 a 13 anos com dados sobre gravidez nessa mesma faixa etária entre 2012 e 2018.

**MÉTODOS:**

estudo epidemiológico, descritivo, de corte transversal, com dados do Departamento de Informática do Sistema Único de Saúde (DATASUS) sobre violência e gestação de meninas com idades entre 10 e 13 anos, dos anos de 2012 a 2018. Os dados sobre violência sexual foram acessados no Sistema de Informação de Agravos de Notificação (SINAN). Os dados sobre gravidez foram buscados em três Sistemas de Informação: o de Nascidos Vivos (SINASC), o de Mortalidade (SIM), para os óbitos fetais e o de Internações hospitalares (SIH), para os de aborto.

**RESULTADOS:**

No período de 2012 a 2018 houve 136.387 gestações, sendo 120.185 nascimentos e 15.402 gestações interrompidas por aborto ou óbito fetal de mães que engravidaram com 13 anos ou menos. No mesmo período foram notificados ao SINAN 46.548 casos de abuso sexual de meninas com idades entre 10 e 13 anos. O número de meninas que engravidaram antes dos 14 anos, vítimas de estupro de vulnerável, foi 2,9 vezes maior do que o número de casos notificados ao SINAN.

**CONCLUSÃO:**

A falta de registro adequado do estupro de vulnerável nas estatísticas oficiais no Brasil leva a subestimativa de sua magnitude.

## INTRODUÇÃO

A violência sexual é um crime de grande proporção no Brasil, porém é pouco evidenciada nas estatísticas oficiais: segundo a última pesquisa nacional de vitimização, apenas 10% das vítimas comunicam a agressão às autoridades policiais^1^ e estima-se que a notificação ao SINAN seja três vezes menor^2^. Meninas menores de 14 anos são as vítimas mais frequentes de ofensas sexuais, do total de casos notificados em 2017 e 2018, mais da metade (53,6%) foram cometidos contra menores de 14 anos, a maioria (81,8%) do sexo feminino, principalmente com idade entre 10 e 13 anos. A situação se agravou em 2019, quando foram registrados 66.348 boletins de ocorrência de estupro e 57,9% das vítimas tinham até 13 anos. Dentre estas, 85,7% eram do sexo feminino^3^, revelando um crescimento de 8% em relação aos dados anteriores e demonstrando que quatro meninas (até 13 anos) são estupradas por hora no Brasil^2^.

Vários estudos corroboram as estatísticas oficiais, evidenciando ainda que, dentre as vítimas de estupro, as meninas negras nessa mesma faixa etária são as que mais sofrem esse tipo de violência^4,5^. Alguns fatores são significativos para essa estatística, como desigualdade de gênero resultante da cultura patriarcal e as vulnerabilidades inerentes a esse período da vida, como dependência econômica, autonomia reduzida, baixa escolaridade e crescimento e desenvolvimento ainda em curso^6^. Em locais onde a desigualdade social e pobreza são maiores, a exploração sexual de menores é um fator importante que incide sobre os índices de violência sexual^9,10^.

A legislação penal brasileira desde 2009 tipifica como crime de estupro de vulnerável ter conjunção carnal ou outro ato libidinoso praticado com menor de 14 anos^11^ e, mesmo a violência sexual sendo um agravo de notificação compulsória, é importante ressaltar que as notificações dessas ofensas sexuais são baixas^1^. A violência sexual muitas vezes não é sequer percebida, muito menos informada, pois acontece com mais frequência dentro do próprio lar, mediante sedução, coerção e/ou ameaças, cometida por familiar ou pessoa conhecida^2,12^, o que ajuda a compreender a subnotificação e a constatar que os índices oficiais de casos notificados não retratam a realidade da violência sexual contra adolescentes nessa faixa etária. Outras violências podem ainda ser menos visibilizadas pela ausência de notificações, como, por exemplo, a violência sexual conjugal^15^.

Os motivos apontados para a não informação dos crimes de violência são o medo de retaliação do agressor, o julgamento após a denúncia, o descrédito nas instituições etc.^2^. Há ainda a hipótese de que a subnotificação dos estupros de vulnerável ocorra por não ser considerada uma violência tanto pela vítima quanto por sua família^16^, que leva à sua normalização caso seja relacionada à tendência de baixa da idade com que adolescentes iniciam a vida sexual na sociedade brasileira, sendo comum que ocorra antes dos 14 anos^17,18^, vide as altas taxas de gestação na faixa etária adolescente. Estes índices, mesmo que estejam reduzindo nos últimos anos, continuam altos em comparação aos países desenvolvidos^19^ e, na faixa etária de 10 a 13 anos, permanecem em ascensão nas regiões Norte e Nordeste do país^20^.

Em vista desses dados, ressalta-se que essas meninas de 10 a 13 anos grávidas são, segundo a lei, vítimas de estupro, e isso pode estar passando à margem das estatísticas oficiais. Uma vez que o crime ocorre principalmente em ambiente privado, alguns fatores concorrem para que a violência não seja notificada, como decisão da família de abafar o crime, situação de vulnerabilidade da vítima ou ainda dificuldade de acesso a serviço público de aborto legal^21^, o que significa que essas gestações vão a termo sem engrossarem as estatísticas oficiais sobre violência sexual.

O presente estudo tem como objetivo comparar dados oficiais de notificação de violência sexual contra meninas com idade entre 10 e 13 anos com dados sobre gravidez na mesma faixa etária, o que corresponde ao estupro de vulnerável, no período entre 2012 e 2018. Pretende-se contribuir para a redução da lacuna de informação sobre a dimensão do estupro de vulnerável no Brasil e, consequentemente, com a implementação de medidas de proteção às vítimas em potencial.

## MÉTODOS

Trata-se de um estudo epidemiológico, descritivo, de corte transversal, desenvolvido com dados do DATASUS sobre violência sexual e gestação, de meninas de 10 a 13 anos, no período de 2012 a 2018. Os dados sobre violência sexual foram acessados nas bases do SINAN de violência^a^, já os dados sobre gestação em adolescentes foram obtidas por meio de informações do Sistema de Informação de Nascidos Vivos (SINASC)^b^, do Sistema de Informações sobre Mortalidade (SIM)^c^ para os óbitos fetais e do Sistema de Internações Hospitalares do SUS (SIH), Capítulo XV do CID-10 (Gravidez, parto e puerpério) e Lista de morbidade – Aborto espontâneo, Aborto por razões médicas e Outras gravidezes que terminam em aborto, para os casos de aborto^d^.

O DATASUS é gerenciado pela Secretaria de Vigilância em Saúde, junto às secretarias estaduais e municipais de Saúde, e os dados são agrupados por faixa etária de 10 a 14 anos. Essas instituições coletam as Declarações de Nascidos Vivos (DNVs) de serviços de saúde e cartórios (para partos domiciliares) e inserem os dados no SINASC. Para estimarmos os dados de 10 a 13 anos foram feitas as seguintes buscas e cálculos. Para cada ano e Unidade Federativa é fornecido um arquivo no formato *dBase File Compacted* (*.dbc), que foi convertido para *dBase File* (*.dbf) via *batch script* (.bat) utilizando o aplicativo TabWin, desenvolvido pelo DATASUS^e^. Uma vez que a base de dados reunida possui 68,8 milhões de registros entre 1994 e 2018, foi necessário utilizar o Database Management System (DBMS) para analisar e manipular o grande volume de dados. Optou-se pelo DBMS opensource PostgreSQL, na versão 11.8. Os dados foram importados para o PostgreSQL a partir dos arquivos DBF por meio de scripts desenvolvidos em linguagem Python na versão 3.8. Como o SINASC registra, a priori, todos os nascidos vivos (NV) do território brasileiro, foram selecionados dois grupos de registros da base de dados para o presente estudo:

Grupo 1: as gestantes com idade entre 10 e 13 anos que tiveram NV no período de 2012 a 2018. A seleção ocorreu por meio da idade da mãe na data do nascimento do filho.

Grupo 2: as adolescentes que começaram a gestação aos 13 anos, porém tiveram o parto do NV já aos 14 anos de idade, no período entre 2012 e 2018. Tal seleção ocorreu pelo cálculo da diferença da idade da mãe na data do nascimento e a quantidade de semanas de gestação ao nascimento.

Apesar da base de dados disponibilizada pelo SINASC no momento deste estudo ser de 1994 a 2018, contabilizou-se no Grupo 1 apenas os registros a partir de 2012, uma vez que entre 1994 e 1995 tal sistema estava em fase de testes e em implantação no Brasil^22,23^. No mais, o cadastro do SINASC teve seus campos alterados ao longo do tempo, onde provavelmente a mudança mais substancial ocorreu entre os anos de 2010 e 2012^f^. A partir do ano de 2012, mais de 90% dos registros de NV passaram a ter o atributo *semagestac* para se calcular a idade da mãe no início da gravidez para o Grupo 2. Por esse motivo selecionou-se o período de 2012 a 2018.

A soma das ocorrências do Grupo 1 com o Grupo 2 resulta na quantidade de registros no SINASC que caracterizam estupro de vulnerável, menor de 14 anos segundo o Art. 217-A da Lei Federal n^o^ 12.015 de 7 de agosto de 2009^11^. Foi calculada a taxa de fecundidade por idade específica (TFIE‰), por meio do número de NV de mães que engravidaram entre 10 e 13 anos por mil adolescentes femininas na mesma faixa etária, por região brasileira.

Os dados disponibilizados pelo SINAN, SIM e SIH também se referem à faixa etária de 10 a 14 anos. Para obtermos uma estimativa do número de casos de violência, de óbitos fetais e de aborto na faixa etária de 10 a 13 anos usamos a projeção do Censo populacional do Instituto Brasileiro de Geografia (IBGE)^24^. Calculamos a proporção da população do sexo feminino com idade entre 10 e 13 anos do total na faixa de 10 a 14 anos do mesmo sexo, e encontramos o valor de 0.79 para todos os anos de 2012 a 2018. Ou seja, a população do sexo feminino com idade entre 10 e 13 anos corresponde a 79% do total da faixa etária entre 10 e 14 anos do sexo feminino. Portanto, estimamos o número de óbitos fetais, de abortos e de notificações de violência sexual de 10 a 13 anos, multiplicando o valor total de 10 a 14 anos por 0,79.

## RESULTADOS

No período entre 2012 e 2018, foram notificados ao SINAN 58.922 casos de violência sexual contra vítimas do sexo feminino com idade entre 10 e 14 anos, sendo a estimativa calculada em 46.548 casos contra meninas com idade entre 10 e 13 anos. Observa-se que neste período houve um aumento gradativo de notificações de casos em todas as regiões brasileiras, apesar de pequena redução em algumas delas no ano de 2015. O Sudeste é a região com maior número de notificações. Contudo, ao se calcular as taxas de violência sexual pela população de cada região, observa-se que em todos os anos a região Norte foi a de maior índice de notificação por 100.000 habitantes do sexo feminino de 10 a 13 anos e a região Sul, a segunda maior. (Tabela 1).

**Tabela 1:** Distribuição do número de casos de violência sexuala notificados pelo SINAN e da taxa por 100.000 habitantes no sexo feminino, na faixa etária de 10 a 13 anos, por Região brasileira notificadas ao SINAN no período de 2012 a 2018.

Ano	Norte n (Tx/100.000)	Nordeste n (Tx/100.000)	Centro-Oeste n (Tx/100.000)	Sudeste n (Tx/100.000)	Sul n (Tx/100.000)
2012	1.164 (169,48)	713 (35,66)	421 (87,42)	1.457 (57,87)	875 (101,63)
2013	1.508 (221,83)	1.084 (55,48)	519 (109,09)	1.555 (63,49)	1.070 (128,52)
2014	1.695 (252,17)	1.285 (67,31)	507 (107,73)	1.615 (67,67)	1.109 (137,33)
2015	1560 (234,28)	1.127 (60,19)	615 (131,41)	1.683 (72,05)	1.139 (144,41)
2016	1671 (252,65)	1.208 (65,54)	684 (146,57)	2.004 (87,68)	1.243 (161,54)
2017	1778 (270,01)	1.427 (78,56)	811 (174,76)	2.538 (112,83)	1.458 (193,33)
2018	1922 (292,67)	1.699 (95,03)	880 (191,18)	2.782 (125,23)	1.741 (234,43)
Total	11.298 (241,35)	8.543 (64,81)	4.437 (134,98)	13.634 (82,91)	8.635 (155,42)

^a^ Na ficha de notificação compulsória dos casos de violência ao SINAN, os seguintes tipos de violência são classificados como violência sexual: assédio sexual, estupro, atentado violento ao pudor, pornografia infantil, exploração sexual e outros.

O número total de nascidos vivos de mães com idade entre 10 e 14 anos no período recortado foi de 178.622, segundo o DATASUS, com redução gradativa ano a ano. Em relação às meninas com idade entre 10 e 13 anos que engravidaram no mesmo período, a Tabela 2 apresenta o número de nascidos vivos do Grupo 1, que inclui somente os NV de mães com menos de 14 anos no momento do parto, o Grupo 2, de mães que engravidaram aos 13 anos, mas tiveram o parto aos 14 anos, e o Grupo 1 adicionado ao Grupo 2, que corresponde a todas que conceberam com 13 anos ou menos. Vale destacar a redução da estimativa do número anual de gestações entre 10 e 13 anos, de 18.348 em 2012 para 14.396 em 2018.

**Tabela 2:** Distribuição anual do número de partos de meninas que conceberam entre 10 e 13 anos de 2012–2018.

Ano	Mãe < 14 anos na concepção e parto (Grupo 1)	Mãe < 14 anos somente na concepção (Grupo 2)	Mãe que engravidou entre 10 e 13 anos (Grupo 1 + 2)
2012	6.671	11.677	18.348
2013	6.491	12.417	18.908
2014	5.832	13.008	18.840
2015	5.828	12.180	18.008
2016	5.565	10.790	16.355
2017	4.986	10.244	15.230
2018	4.882	9.614	14.496
Total	40.255	79.930	120.185

A Tabela 3 apresenta o nº total de nascidos vivos/TFIE‰ de todas as meninas que conceberam quando tinham entre 10 e 13 anos (Grupo 1 e Grupo 2), distribuídos por regiões brasileiras. As maiores taxas ocorreram nas regiões Norte e Nordeste. Quando se comparam os anos iniciais da série com os últimos, observa-se que o padrão de redução anual dos NV de mães que conceberam quando tinham entre 10 e 13 anos ocorreu em todas as regiões, com exceção de discreto aumento em 2018 na região Norte.

**Tabela 3:** Distribuição do número de NV de meninas que engravidaram entre 10 e 13 anos (Grupo 1 + Grupo 2) e da TFIE‰a por Região brasileira no período de 2012 a 2018.

Ano	Norte n (TFIE‰)	Nordeste n (TFIE‰)	Centro-Oeste n (TFIE‰)	Sudeste n (TFIE‰)	Sul n (TFIE‰)
2012	3.280 (4,88)	6.962 (4,12)	1.613 (3,2)	4.695 (1,87)	1.798 (2,09)
2013	3.446 (5,18)	7.032 (4,25)	1.660 (3,34)	4.947 (2,03)	1.823 (2,2)
2014	3.321 (5,05)	6.882 (4,24)	1.693 (3,44)	5.054 (2,12)	1.890 (2,35)
2015	3.248 (5,0)	6.864 (4,3)	1.576 (3,2)	4.651 (2,0)	1.669 (2,13)
2016	3.132 (4,83)	6.223 (3,94)	1.488 (3,02)	4.077 (1,77)	1.435 (1,86)
2017	2.874 (4,45)	5.942 (3,82)	1.362 (2,79)	3.802 (1,68)	1.250 (1,65)
2018	2.914 (4,52)	5.714 (3,74)	1.272 (2,63)	3.506 (1,57)	1.090 (1,46)
Total	22.215 (4,84)	45.619 (4,05)	10.664 (3,08)	30.732 (1,86)	10.955 (1,96)

^a^ Nascidos vivos (NV) de mães que engravidaram entre 10 e 13 anos por mil adolescentes femininas da mesma faixa etária.

Quanto às gravidezes interrompidas, o número total de óbitos fetais e de abortamentos hospitalares (por razões médicas, espontâneo e por outras causas) de meninas de 10 a 14 anos no período de 2012 a 2018, segundo o SIM e SIH, foi de 19.497 (3.290, 3.167, 3.282, 2.824, 2.545, 2.364, 2.025, respectivamente). A estimativa do total de gestações interrompidas em meninas de 10 a 13 anos é de 15.402 no mesmo período. A distribuição por ano pode ser visualizada na Tabela 4.

**Tabela 4:** Razão de notificação e distribuição anual dos casos de estupro de vulnerável e das notificações registradas no SINAN no Brasil de 2012 a 2018.

Ano	Gestações de 10 a 13 anos com NV	Abortos e óbitos fetais entre 10 e 13^a^	Total estupros vulneráveis	Notificações de VS em meninas de 10 e 13^a^	Razão^a^
2012	18.348	2.599	20.947	4.631	4,5
2013	18.908	2.502	21.410	5.736	3,7
2014	18.840	2.593	21.433	6.211	3,5
2015	18.008	2.231	21.039	6.124	3,4
2016	16.355	2.010	18.365	6.810	2,7
2017	15.230	1.867	17.097	8.012	2,1
2018	14.496	1.600	16.096	9.024	1,8
**TOTAL**	**120.185**	**15.402**	**135.587**	**46.548**	**2,9**

^a^ Razão entre número de gestações em meninas de 10 a 13 anos (estupros de vulneráveis) e o número de casos de violência sexual (VS) notificados na mesma faixa etária no sexo feminino.

A Tabela 4 descreve a estimativa de estupros de vulneráveis e sua relação com os casos notificados no SINAN. São apresentados os dados de NV de mães que engravidaram antes dos 14 anos, o número de gestações interrompidas, a soma destes dois números que corresponde ao total de meninas que engravidaram antes dos 14 anos, tendo a gestação sido interrompida ou levada a termo, ou seja, total das que foram vítimas de estupro de vulnerável, segundo o código penal, e número de notificações de violência sexual (VS) contra o sexo feminino também ajustado para a faixa etária de 10 a 13 anos). Na última coluna, evidencia-se a razão da não notificação do caso de estupro de vulnerável. Vale salientar que esta razão de chance deve estar extremamente subnotificada, pois grande número de casos de violência que não resultam em gravidez não são notificados ou por vezes nem mesmo reconhecidos como tal pela vítima ou seus responsáveis.

## DISCUSSÃO

Este estudo demonstra que as estatísticas oficiais não evidenciam a amplitude do estupro de vulnerável no país, pois somente o quantitativo de gestações por meninas com idade entre 10 e 13 anos foi quase três vezes maior do que os casos de violência sexual em meninas na mesma faixa etária notificados ao setor de saúde nos anos entre 2012 e 2018. Os resultados encontrados, contudo, são menos alarmantes do que o verificado em estudo comparativo entre adolescentes gestantes menores de 14 anos, cuja violência foi notificada aos órgãos competentes, e aquelas que não foram notificadas, no período de 2011 a 2015. Do total de 31.611 nascidos vivos de mães com até 13 anos, foram registrados no SINAN apenas 4,3%^12^.

Os registros do SINAN mostram um aumento do número de notificações, em todas as regiões ao longo dos anos estudados, o que pode evidenciar uma ascensão deste grave problema de saúde pública e/ou uma melhora no sistema de notificação. A região Norte foi a de maior índice de notificação de violência sexual contra meninas com idade entre 10 e 13 anos em todos os anos e a região Sul a segunda maior. Contudo, vale registrar que a (sub)notificação dos casos de violência sexual não deve ser equivalente em todas as regiões do Brasil, pois o Nordeste apresentou a segunda maior média TFIE em idade de 10 a 13 anos no período de 2012 a 2018, só atrás da região Norte, e é a com menor taxa de notificação de violência sexual.

Estudo realizado por Gaspar et al (2018)^25^, sobre a evolução da notificação de violência sexual no Brasil entre 2009 e 2013 demonstrou avanço principalmente dos casos de estupro, de estupro dentro de casa e da violência sexual de repetição. Os autores consideram que houve acréscimo real do número de casos e ampliação da notificação, devido à maior conscientização, tanto das vítimas em procurar atendimento quanto dos profissionais que as atendem, e que também houve melhora no sistema de notificação. Outro dado digno de nota é a mudança do código penal, em 2009, que tipificou o estupro de vulnerável. A partir daí um conjunto maior de ações mobilizou o governo e a sociedade para o enfrentamento desse problema, o que pode ter influenciado positivamente no sistema de notificação^26^. Por outro lado, a subnotificação foi evidenciada em outro estudo, realizado por meio de registros de nascidos vivos de mães de 10 a 13 anos no nordeste brasileiro. As autoras verificaram que apenas 1,3% foram notificadas como vítimas de abuso sexual^27^.

As gravidezes em menores de 14 anos que, aos olhos da lei, tratam-se de casos de estupro de vulnerável e a subnotificação dos mesmos ao SINAN revela a face oculta das estatísticas de violência sexual no Brasil, sem levar em conta outras violências sexuais nesta faixa etária que não resultam em gravidez e provavelmente também não são notificadas. Ao refletir sobre o resultado e o porquê da subnotificação, uma das hipóteses é que essas gestações possam ser fruto de relações sexuais consentidas em que as adolescentes e suas famílias não consideram que foram violentadas. Sabe-se que um percentual considerável de adolescentes menores de 14 anos tem prática sexual e, muitas vezes, com o conhecimento e aprovação da família e que, por vezes, resultam em casamento. Estudo realizado com estudantes do Ensino Médio sobre a percepção da iniciação e da violência sexual evidenciou uma desconexão entre o que pensam os adolescentes e o que é previsto na lei. Há uma necessidade de ampliação do debate sobre o tema, com participação do público-alvo, acompanhado de intensificação nos programas de educação sexual para jovens^16^. Outro dado digno de nota é o fato de frequentemente o agressor ser um familiar ou conhecido da família, portanto, dificultando a notificação tanto pela família quanto pelo profissional de saúde^2,14^.

Vale destacar a importância da notificação dos casos de estupro de vulnerável para proteção das vítimas e a tomada de medidas judiciais cabíveis para punição dos agressores. É obrigação legal do profissional de saúde notificar, porém, ocasionalmente este entende que não deve fazê-lo, pois avalia que o relacionamento sexual foi consentido ou enfrenta barreiras nos locais onde não há rede de apoio bem estruturada. Outro dado que contribui com a não notificação é o medo do profissional de sofrer retaliações por parte do agressor, pois, por vezes, esse registro é encarado como denúncia e não como uma iniciativa para proteção da vítima^16,27^. Portanto, fica evidente a importância da capacitação desses profissionais para lidar com o tema, incluindo o manejo das relações familiares e a notificação ao conselho tutelar.

A violência sexual é uma questão de saúde pública e de violação dos direitos humanos. É notório que as consequências de um estupro para a vida da adolescente são graves e múltiplas. De imediato é necessária a profilaxia de infecções sexualmente transmissíveis, HIV e prevenção da gravidez. A médio e longo prazo sobressaem os problemas relacionados à saúde mental, autoestima, sociabilidade e crescimento e desenvolvimento^28^. É também comum a reincidência da violência, maior risco de consumo de bebidas alcoólicas e de drogas, de atividade sexual sem proteção e de exploração sexual comercial^29^.

Por último, ressalta-se que sendo a gestação antes dos 14 anos tipificada como crime, ela dá direito à vítima de interrupção legal da gravidez. Todavia, estudos e acontecimento recente, de uma menina de 10 anos grávida consequente de estupro perpetrado por um tio, noticiada em rede nacional, demonstram a dificuldade dessas meninas em ter esse direito assegurado, de ser reconhecida como vítima de violência sexual^30,31^. Esse caso é emblemático, pois evidencia uma barreira a mais na proteção das vítimas de violência sexual no Brasil.

Este estudo é limitado, pois utiliza somente dados secundários e restritos a meninas que engravidaram antes dos 14 anos, mas traz a contribuição de dar visibilidade a esses dados. Pode-se inferir que o número de meninas menores de 14 anos que sofrem violência sexual é bem maior, pois nem as que são atendidas em hospitais foram amiúde notificadas. Além disso, o Atlas da Violência confirma que menos da metade das vítimas que registram a ocorrência policial procuram atendimento nas unidades de saúde^2^. Outros setores responsáveis pela proteção a crianças e adolescentes deveriam se envolver de forma mais efetiva na estrutura de assistência às adolescentes vítimas de violência e na redução do sub-registro e da própria violência, como a área da Educação, os conselhos tutelares, a Defensoria e o Ministério Público.

Concluímos que a não notificação dos casos de estupro de vulnerável é uma das faces deste grave e crônico problema de saúde pública. A falta de registro adequado do estupro de vulnerável nas estatísticas oficiais no Brasil leva à subestimativa de sua magnitude. São necessárias políticas públicas que aperfeiçoem o sistema de notificação de agravos de violência sexual e as garantias de direito à proteção das vítimas, principalmente as que estão em posição de maior vulnerabilidade individual e social e com menor acesso aos serviços de saúde, como as meninas menores de 14 anos.

## Footnotes

a Ministério da Saúde (BR), DATASUS –Notificação de violência sexual sexo feminino de 10 a 14 anos. Brasília, DF [citado 13 set 2020]. Disponível em: http://tabnet.datasus.gov.br/cgi/tabcgi.exe?sinannet/violencia/bases/violebrnet.def

b Ministério da Saúde (BR), DATASUS. Transferência de Arquivos do SINASC. Brasília, DF [citado 13 jul. 2020]. Disponível em: http://www2.datasus.gov.br/DATASUS/index.php?area=0901&item=1&acao=28&pad=31655

c Ministério da Saúde (BR), DATASUS. Informações de Saúde (TABNET). Estatísticas vitais: óbitos fetais. Brasília, DF [citado 31 out 2020]. Disponível em: http://tabnet.datasus.gov.br/cgi/deftohtm.exe?sim/cnv/fet10uf.def

d Ministério da Saúde (BR), DATASUS. Morbidade Hospitalar do SUS – por local de residência – Internações por Ano de atendimento segundo Região. Lista Morb CID-10: Outras gravidezes que terminam em aborto. Faixa etária 1: 10 a 14 anos. Período: 2012. [citado 31 out 2020]. Disponível em: http://tabnet.datasus.gov.br/cgi/tabcgi.exe?sih/cnv/nruf.def

e Ministério da Saúde (BR), DATASUS. Transferência de Arquivos do SINASC. Brasília, DF [citado 13 jul 2020]. Disponível em: http://www2.datasus.gov.br/DATASUS/index.php?area=060805&item=3

f Ministério da Saúde (BR), Secretaria de Vigilância em Saúde, Coordenação Geral de Informações e Análises Epidemiológicas. Consolidação do Sistema de Informações Sobre Nascidos Vivos – 2011. Brasília, DF; 2013 [citado 13 jul 2020]. Disponível em: http://tabnet.datasus.gov.br/cgi/sinasc/Consolida_Sinasc_2011.pdf
